# Hepatovirus infections in juvenile seals from the North Sea

**DOI:** 10.1038/s44298-024-00084-8

**Published:** 2025-01-08

**Authors:** Monica Mirolo, Bianca Kühl, Melvin Daniel Roji, Ana Rubio-García, Valéria Andrade Lima, Christina Puff, Byron Martina, Andreas Beineke, Peter Wohlsein, Wolfgang Baumgärtner, Martin Ludlow, Albert Osterhaus

**Affiliations:** 1 Research Center for Emerging Infections and Zoonoses, University of Veterinary Medicine Hannover, Foundation, Hannover, Germany; 2 Department of Pathology, University of Veterinary Medicine Hannover, Foundation, Hannover, Germany; 3 Sealcentre Pieterburen, Pieterburen, The Netherlands; 4 Artemis Bioservices, Delft, The Netherlands

**Keywords:** Virology, Epidemiology

## Abstract

The discovery of several novel hepatovirus species in marine and terrestrial mammals has expanded the recognised members of the genus *Hepatovirus* and has provided better understanding on the evolutionary origins of human hepatovirus A (HAV). Using high throughput sequencing we detected a seal hepatovirus (SealHAV_NL/PV/21), in liver tissue of a deceased harbor seal (*Phoca vitulina*) originating from the Dutch North Sea coast. RT-PCR screening of liver samples of 88 harbor seals and 12 grey seals (*Halichoerus grypus*) from the same region identified seal hepatovirus in nine juvenile harbor seals in which minor sequence variation was observed in the VP1 gene. Whole-genome sequence analysis showed that SealHAV_NL/PV/21 displayed 95.6% nucleotide indentity to New England seal hepatovirus but had a 5′-UTR which contained additional 51 bp. Phylogenetic analysis showed that seal hepatoviruses clustered in a monophyletic group separate from other hepatovirus species that have been identified in terrestrial mammals. Assessment of seal hepatovirus RNA loads in organs of all infected animals showed that the liver had the highest number of RNA copies with up to 10^7^ RNA copies per mg of tissue. Seal hepatovirus RNA was readily detected by in situ hybridization in hepatocytes in the liver but was not associated with pathological lesions. Serological screening of 90 contemporary seal sera using a HAV-based ELISA showed the presence of hepatovirus antibodies in 14 harbor seals and one juvenile grey seal. These findings collectively show that seal hepatovirus is enzootic among seals of the North Sea, causing quiescent infections in young animals.

## Introduction

The identification of novel hepatovirus species related to human hepatovirus A (HAV) in a wide variety of mammalian species has rapidly increased in recent years. This has generated new insights into the evolutionary history of HAV, which remains an important cause of human hepatitis worldwide. Hepatoviruses are classified within the genus *Hepatovirus* of the family *Picornaviridae* and contain a positive-sense, single-stranded RNA genome of ~7500 bp. The genome contains a single open reading frame (ORF) organized into three functional regions, P1, P2 and P3^1^. The proteolytic cleavage of the P1, P2, and P3 regions results in the production of the viral structural and non-structural proteins. The P1 region encodes the capsid polypeptides VP1, VP2, VP3, and VP4, while the P2 and P3 regions encode non-structural proteins involved in virus replication and capsid formation: 2A, 2B, 2C, 3A, 3B, 3Cpro, and 3Dpol^2–4^. Hepatovirus-specific antibodies are predominantly directed against defined immunodominant antigenic sites, largely comprised of amino acid residues in the VP1-3 proteins^5^.

Non-human primate hepatoviruses and HAV genotypes constituted the entire known genetic diversity of HAV until the identification in 2015 of an additional 13 hepatoviruses in 209 species of small mammals from five different mammalian orders: *Rodentia* (rodents), *Scandentia* (treeshrews), *Chiroptera* (bats), *Eulipotyphla* (hedgehogs, shrews), and *Afrosoricida* collected in European, Asian, African, and American countries^6^. This provided new insights into the potential evolutionary origins of HAV. Subsequently, further research in wild and domestic animals resulted in the discovery of hepatoviruses in blood and organs of opossums (*Didelphis aurita*), and blood and faeces of alpacas (*Vicugna pacos*) in South America^7,8^. In addition to the discovery of hepatovirus species in terrestrial wildlife, a novel hepatovirus (Hepatovirus B1), was identified in harbor seals (*Phoca vitulina*) and a harp seal (*Pagophilus groenlandicus*) on the New England coast in 2015^9^. Molecular and phylogenetic analyses demonstrated a close evolutionary relationship between New England seal hepatovirus and HAV.

In this study, we have characterized a seal hepatovirus strain, named SealHAV_NL/PV/21, in harbor seals from coastal waters of the Netherlands. Molecular, serological, and pathological data indicate that this virus is enzootic among seals in this region and can cause quiescent infections in young seals.

## Materials and methods

### Ethical statements

The handling and sampling of wild seals at the Sealcentre Pieterburen was performed with the permission of the government of the Netherlands (number FF/75/2012/015). All pathological and virological analyses at the University of Veterinary Medicine Hannover were carried out under permit number DE 03 201 0043 21 obtained from the Fachbereich Öffentliche Ordnung, Gewerbe- und Veterinärangelegenheiten, Hannover, Germany.

### Samples

The Sealcentre Pieterburen, the Netherlands, provided tissue samples (lung, brain, spleen, kidney, and liver) from a 6-month-old male harbor seal (PV21010601), which had stranded and then died ten days post-admission in January 2021. The necropsy was performed at the Sealcentre Pieterburen. Tissue samples were stored at −80 °C for virological and molecular analyses or fixed in 10% buffered formalin for patho-histological analyses. Frozen samples and samples in formalin were transported to the University of Veterinary Medicine Hannover, Germany. Additional postmortem samples (lung, brain, spleen, kidney, liver) from 88 harbor seals and 12 grey seals necropsied at the Sealcentre Pieterburen and stored at −80 °C or fixed in 10% buffered formalin, were also provided by the Sealcentre for further virological, and pathology analyses. Details on all screened seals, and carcass preservation status at the necropsy are illustrated in Table S1. Of these 100 seals necropsied, some were found dead on the coast or died in the Sealcentre during rehabilitation, while for other seals euthanasia was required. A licenced veterinarian performed the euthanasia by sedating the animal with ketamine (15 mg/kg) and diazepam (0.1 mg/kg) intravenously and then injecting pentobarbital sodium (100 mg/kg) intravenously. In addition, 90 serum samples which had been collected from this seal cohort in 2020 and 2021 were also provided by the Sealcentre Pieterburen for serological testing.

### Sample processing and high throughput sequencing

A Lver tissue from a harbor seal (ID number: PV21010601) was processed for high throughput sequencing (HTS) as described previously^10^. Briefly, a 20 mg tissue section from liver was lysed in 500 μL of PBS in a FastPrep-24 5G homogenizer (MP Biomedical). Homogenates were centrifuged, and RNA was isolated from 250 supernatant μL using TRIzol (Thermo Fischer Scientifics, Waltham, MA, USA) as per manufacturer instructions, and transcribed to cDNA using a mix of random and non-ribosomal hexamers^11^ by Superscript IV (Thermo Fischer Scientifics). Klenow fragment (New England Bio-lab [NEB], Ipswich, MA, USA) was used to produce the second cDNA strand. Random amplification of DNA samples was performed using a sequence-independent, single-primer amplification protocol^12^. The PCR products were purified, and DNA library preparation was performed using a Nextera XT DNA Library Preparation Kit (Illumina, San Diego, CA, USA) prior to sequencing on a NextSeq 550 platform. Initial bioinformatics analysis from raw FASTQ sequencing data was carried out using the CZ ID pipeline^13,14^. Viral genome termini were sequenced using rapid amplification of cDNA ends (RACE) with a 5′/3′ RACE Kit (Roche) using primers RACE1 5′-GCTAAGGCGTCACTTATG-3′, and RACE2 5′-GTGTGCTTCAATAGTCCAG-3′ for the 5′-end, and RACE3 5′-CGCATTTATGCATGGAATGC-3′, and RACE4 5′-GCAAGAATGGCAAGAATTTCTG-3′ for the 3′-end.

### Screening of tissue samples by RT-PCR

RNA was extracted from approximately 60 mg of liver tissue samples from 88 harbor seals and 12 grey seals necropsied between 23rd February 2020 and 9th December 2021 using a KingFisher Flex Purification System (Thermo Fisher Scientific, Switzerland) with the NucleoMag RNA Kit (Machery Nagel, Switzerland) as per manufacturer’s instructions. Reverse Transcription Polymerase Chain Reaction (RT-PCR) was performed with a OneStep RT-PCR Kit (Qiagen) using primers spanning the conserved 5′-UTR including the newly designed forward primer 5′-CCGTGGTTTACGGCTACC-3′, and the reverse primer RACE2 at an annealing temperature of 46 °C. The genetic variation of seal hepatovirus present in positive samples was investigated by sequencing the VP1 gene. RNA extracted from these positive samples was reverse transcribed into cDNA. A PCR was performed with Q5 High-fidelity DNA polymerase (NEB) kit using primers VP1 frw 5′-GGTTAAGTCATCCTAGCAATG-3′ and VP1 rvs 5′-CCTCCTGTGTTAGCTGTTC-3′ at an annealing temperature of 62 °C. The 1039 bp amplicons were analysed by gel electrophoresis, purified using the Monarch® DNA Gel Extraction Kit (New England BioLabs), and Sanger sequenced. Samples that tested positive for hepatovirus in liver tissue were also tested for the presence of phocine herpesvirus-1 (PhHV-1), a major cause of hepatitis in young seals^15^, using a previously published primer set^15,16^.

### Validation of the RT-qPCR assay to assess viral RNA loads

Positive-control RNA was generated via in vitro transcription of a cloned 132-bp fragment of Phopivirus VP1 which had been inserted adjacent to a T7 promoter in the pcDNA3.1 vector. Generation of the EGFP internal control has been performed in a previous study^17^. Briefly, the positive DNA control was linearized by Eco-RI restriction enzyme, and RNA was produced using the HiScribe T7 high-yield RNA synthesis kit (NEB) according to manufacturer’s instructions. The RNA yield was determined photometrically from 2 μL of RNA at an absorbance of 260 nm and 280 nm with a Thermo Scientific™ Multiskan™ GO Microplate Spectrophotometer. The copy number of the newly generated RNA was determined using the equation (amount RNA (ng/µl) × 6.022 × 10^23^/(RNA fragment length (bp) × 340) = Y (RNA molecules/µl). The RNA that was generated from positive and GFP internal control was tested in a duplex RT-qPCR assay, which detects simultaneously FAM (positive control) and HEX (hEGFP internal control). Following assay validation, RNA was extracted from multiple organs (spleen, kidney, brain, and lung) from all hepatovirus-positive seals using RNAeasy Mini kit (Qiagen). All RT-qPCRs were performed using the Luna universal probe one-step RT-qPCR kit (NEB) as per the manufacturer’s instructions using a concentration of 0.4 μM for each primer and 0.2 μM for each probe in the RT-qPCR master mix. Assays were performed using a using a LightCycler 96 Real-Time PCR System (Roche) with an initial 10-min RT step at 55 °C, followed by incubation for 1 min at 95 °C. Forty-five two-step cycles were then performed using the following conditions: denaturation at 95 °C for 10 s, annealing and amplification at 60 °C for 30 s. The fluorescence level was detected and quantified. To produce the standard curves, the RNA from the positive control and the heterologous EGFP internal control was 10-fold serially diluted 9 times. Eight replicates of each dilution were tested in the duplex assay. The Cq values for each dilution were plotted against the respective log2 RNA concentrations (copy number/μL) and simple linear regression analysis was performed using GraphPad Prism 8 software to automatically produce slope, intercept, and R^2^ values for standard curves for the positive and heterologous internal EGFP controls. The resulting Cq values were plotted against the logarithm of each respective RNA concentration, starting from a 6.78 × 10^11^ RNA copies/mL positive control, and 5.06 × 10^11^ RNA copies/mL for the EGFP internal control at a 1:10 dilution. The standard curve for the positive control showed a slope of −2.896 and a R^2^ of 0.9931 (Fig. S1A), whereas the standard curve for the heterologous internal EGFP control showed a slope of −3.416 and a R^2^ of 0.9814 (Fig. S1B).

### Assessment of viral RNA loads in seal organs using the newly established RT-qPCR assay

A new quantitative RT-PCR (RT-qPCR) assay was designed and validated to enable quantification of RNA copies of seal hepatovirus in solid organs (liver, lung, spleen, kidney, and brain) of positive seals. A 132 bp region of the VP1 gene was chosen as the target for amplification using the specific primers 5′-TAGAGTCAGTGTGGCAGGTG-3′ (forward), 5′-TGTGTTTTCTTTGAGCCGGG-3′ (reverse), and 5′-[FAM]CTTCTGTTGCCGGAACTCATCAAGATGAAA [BHQ1]-3′ (probe). The RNA copy number was calculated from a standard curve that had been generated by performing this RT-qPCR assay on RNA generated via in vitro transcription of a cloned 132 bp region of VP1 corresponding to the amplicon generated by the forward and reverse primers.

### Molecular and phylogenetic analyses

Alignment of nucleotide and protein sequences was performed using MAFFT^18^. A consensus maximum likelihood (ML) phylogenetic tree was built in MEGA11^19^ according to the general-time-reversible model with gamma distribution rate variation and a proportion of invariable sites (GTR + G + I). Avian encephalomyelitis virus was used as an outgroup. Sequence analyses, and genome annotations were carried out in Geneious Prime (v2021.0.1, Biomatters, New Zealand).

### Histology and fluorescence in situ hybridization (FISH)

For microscopic examination, the tissues were processed routinely and embedded in paraffin wax with subsequent sectioning at 3 µm thickness, followed by hematoxylin and eosin (H&E) staining. The RNA-FISH probe was produced commercially (ViewRNA Type 1 probe set, Life Technologies GmbH, Darmstadt, Germany) using as a template a 1343 bp genome fragment spanning VP2, VP3, VP4. Tissue sections of liver, lung, kidney, and spleen of Phopivirus positive seals and liver, lung, spleen, kidney, brain of a virus-negative seal, were primed with the probe for 60 min at 60 °C. The next day, tissue sections were deparaffinized, followed by incubation in Pretreatment Solution at 85–90 °C for 20 min, and proteolytic digestion with protease QF® for another 10 min at 40 °C. Sections were then fixed with 4% paraformaldehyde, treated with 0.2 molar hydrochloric acid for another 10 min, and hybridized with the FISH probe for 4 h at 40 °C. Subsequently, pre-amplifier, amplifier, and AP-linked labelled probe were added to perform AP enhancement. Finally, slides were stained using Fast Red Substrate and counterstained with Mayer’s hemalum (Carl Roth GmbH + Co. KG.). FISH was performed using the ViewRNA ISH Tisue Core Kit (Invitrogen by Thermo Fisher Scientific, Vienna, Austria). To check the specificity of the RNA-FISH probe for SealHAV_NL/PV/21, the sequence used to generate the probe was blasted to the host genome (by BLASTn). FISH was then performed with modified manufacturer’s protocol^20,21^. The specificity of the staining was validated by incubating tissue sections from a hepatovirus PCR-negative seal with RNA-FISH probe for SealHAV_NL/PV/21.

#### Serology

A total of 90 serum samples collected routinely from moribund or convalescent harbor and grey seals in the Seal Centre Pieterburen from years 2020 and 2021, were tested for the presence of hepatovirus-specific antibodies, using a commercially available anti-HAV pseudo-competitive ELISA (Mediagnost). These 90 serum samples included 64 sera obtained at rehabilitation day 0 (60 harbor seals; 4 grey seals) and subsequently collected at different time points during the rehabilitation period (26 serum samples from 22 harbor seals and 3 grey seals). All sera were heat-inactivated at 56 °C for 30 min and diluted 1:20 in PBS prior to testing. For statistical analyses, GraphPad Prism software (version 9.0.0, GraphPad Software Inc.) was used. Data were checked for normality by D’Agostino & Pearson test and a non-parametric Mann–Whitney test was used to compare the mean optical density (OD) between samples classified as positive and negative for HAV cross-reactive antibodies. A *P* value < 0.05 was considered significant.

## Results

### Discovery of a new strain of seal hepatovirus in a harbor seal from the North Sea

A stranded 6-month-old harbor seal (PV21010601) had died in January 2021, ten days after admittance to the Sealcentre Pieterburen displaying signs of severe respiratory disease. g necropsy, macroscopic changes in the lungs and the liver suggesting inflammation, were noted. Upon histopathological analysis, the liver tissue showed multifocal hepatic necrosis and intranuclear viral inclusions, indicative of PhHV-1 infection. A previously designed pan-herpesvirus PCR assay^16^ confirmed the presence of a herpesvirus in the seal liver, and Sanger sequencing on the resulting amplicon showed that the recovered herpesvirus sequence was 100% identical to the PhHV-1 strain previously identified in seals from the North Sea coasts^15^. In addition, HTS was also performed on RNA extracted from this liver tissue resulting in the recovery 35,230 trimmed reads with highest similarity to seal hepatovirus NewEngland_USA/2011 (GenBank accession no. NC027818.1), named Phopivirus^9^, spanning nearly the full-length ORF. Genome termini were sequenced using specific primers designed based on available seal hepatovirus sequence recovered by HTS. The full-length genome of the hepatovirus strain identified in this study displayed 95.6% nucleotide identity to the NewEngland_USA/2011 virus and was named “SealHAV_NL/PV/21” (Gen Bank accession number PP145370).

### Genome analyses of SealHAV_NL/PV/21

The genome of SealHAV_NL/PV/21 is 7527 bp with 37.4% GC content, and a structure that corresponded to other members of the genus Hepatovirus: VPg+5′-UTR (IRES-III)-[1A-1B-1C-1D-2A/2B-2C/3A-3B-3C-3D]-3′-UTR-poly(A)^1^. The seal hepatovirus strains from New England and The Netherlands were 99.1% identical at the 5′ UTR, and 93% identical at the 3′UTR. The nucleotide sequence within the 5′ UTR corresponding to the type III internal ribosome entry site (IRES) was identical in both strains. However, the 5′UTR of the SealHAV_NL/PV/2021 genome contained an additional contiguous 51 nucleotides characterized by repetitive base pairs sequences. The single ORF encoded a polyprotein between nucleotide 690 and 7382 of 2230 codons comprising the P1, P2, and P3 functional domains. In comparison to the New England seal hepatovirus strain, amino acid changes in the P2 and P3 regions included two substitutions in each of the 2AB (S902N, I941V), 2C (I1126V, E1363D), and 3D (R1758K, F2108L) proteins, along with a single amino acid change in 3A (D1486N) and 3C (I1625V) proteins (Table 1). No amino acid changes were found in the P1 domain encoding for structural proteins.

**Table 1:** Amino acid changes in non-structural proteins between the New England and Dutch Phopivirus strains

Non-synonymous amino acid substitutions in polyprotein regions of seal hepatovirus SealHAV_NL/PV/21								
	2AB		2C peptide		3A peptide	3C peptide	3D peptide	
Codons^a^	AGU/AAU	AUU/GUU	AUC/GUU	GAA/GAU	GAU/AUU	AUC/GUC	AGA/AAA	UUU/CUU
aa^b^	S902N	I941V	I1126V	E1363D	D1486N	I1625V	R1758K	F2108L

^a^Codons are reported in the following order: NC_027818/SealHAV_NL/PV/21.

^b^Mutations are reported as follows: aa of NC_027818/ position in CDS/ aa of SealHAV_NL/PV/21.

Additional molecular screening for seal hepatovirus was performed by RT-PCR using virus-specific primers on liver samples collected from 88 harbor seals and 12 grey seals from the same geographical region between 2020 and 2021. Only six of these animals were adults, and ninety-five were juveniles. Virus-specific amplicons were detected in liver tissue of an additional nine juvenile harbor seals. No consistent pathomorphological pattern was observed macroscopically in the liver and other organs among the ten positive seals (Table S2). Minor synonymous nucleotide changes were observed in the VP1 gene of the seal hepatovirus strain present in infected animals (Table S3) (GenBank accession numbers PP145360-69).

### Assessment of viral load in solid organs

**Fig. 1:**
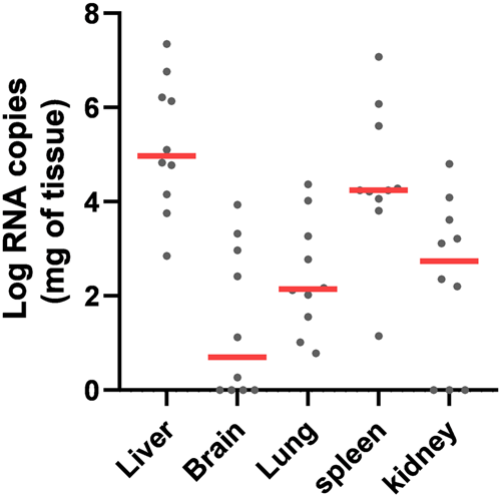
Seal hepatovirus RNA concentrations in solid seal organs. The Log RNA copies per milligram of liver, brain, lung, spleen and kidney tissue samples from seal hepatovirus-positive seals (*n* = 10) are reported as individual values (grey dots) with group mean (solid red lines).

The tissue distribution and level of infection of SealHAV_NL/PV/21 was investigated using a newly designed RT-qPCR assay. The efficiency of this assay was assessed to facilitate quantification of viral RNA in tissue samples from the ten positive animals, by performing a duplex RT-qPCR assay on nine 10-fold dilutions of both positive and heterologous internal EGFP controls. We determined the number of RNA copies of seal hepatovirus in each organ using the seal hepatovirus positive control standard curve (Fig. S1). The liver consistently had the highest number of RNA copies compared to all other organs, with up to 10^7^ RNA copies per mg of tissue, although RNA was also detected in extrahepatic tissues at variable but generally lower average levels (Fig. 1).

### Evolutionary relationship of the SealHAV_NL/PV/21 to other hepatoviruses

**Fig. 2:**
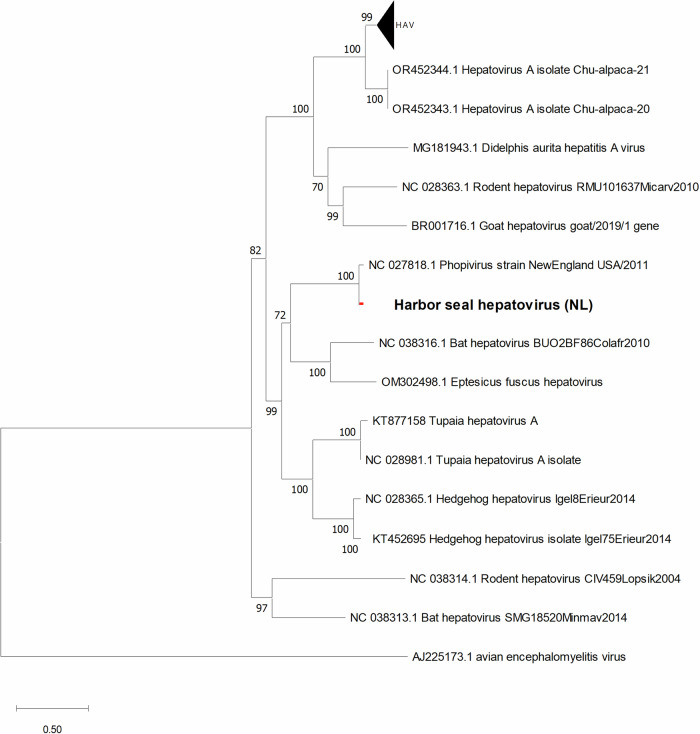
Maximum likelihood phylogenetic tree of consensus full-length sequences of hepatovirus CDS. The ML trees were calculated using GTR + G + I model with 1000 bootstraps. The scale bar indicates the nucleotide substitutions per site. All sequences used in the phylogenetic tree are listed with their GenBank accession numbers and names in Table S4.

Phylogenetic and genome analyses were performed to investigate the evolutionary relationship between SealHAV_NL/PV/21, HAV and other mammalian hepatoviruses. The maximum likelihood phylogenetic tree based on full-length CDS hepatovirus sequences showed that both seal hepatoviruses clustered with hepatoviruses from bats (GenBank accession no. OQ818337 and no. NC_038316) in a monophyletic group with a statistically significant probability (Fig. 2). SealHAV_NL/PV/21 was 73.87% identical to big brown bat (*Epistecus fuscus*) hepatovirus (GenBank accession no. OM302498), 73.99% to another bat hepatovirus (GenBank accession no. NC_038316) and has 71.83% nucleotide identity with HAV (GenBank accession no. ON524442).

### Histological findings and FISH

**Fig. 3:**
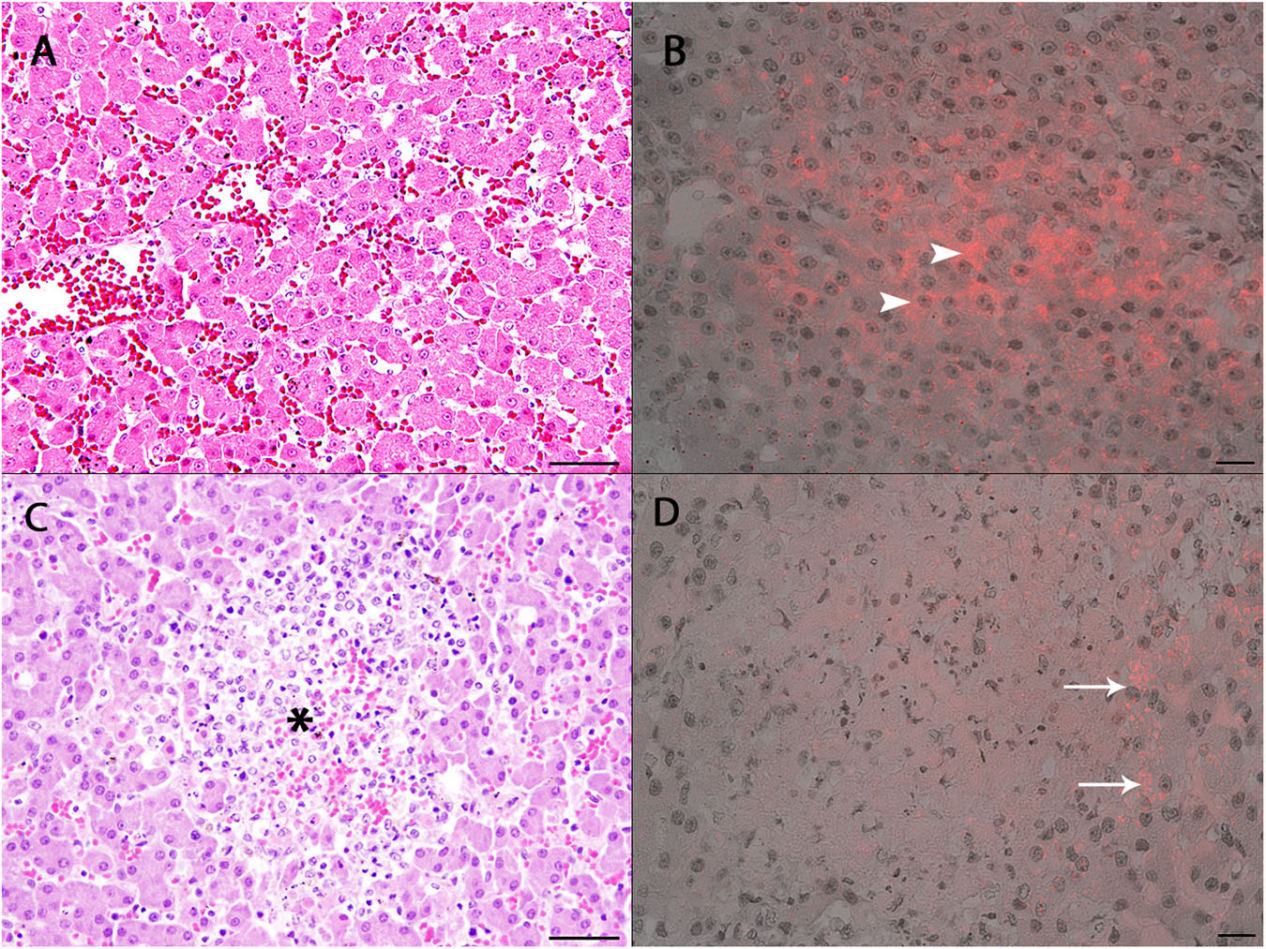
Pathological analysis of seal hepatovirus infected liver tissues. **A** H&E staining. Liver section of seal PV20121801. No inflammation in the liver tissue and presence of morphologically normal hepatocytes. Bar: 50 µm. **B** FISH. Liver section of seal PV20121801. Seal hepatovirus RNA is detected within the cytoplasm of normal hepatocytes (arrowheads). **C** H&E staining. Liver section of seal PV20010801. Focal liver necrosis (asterisk) with accumulation of debris and granulocytic and histiocytic infiltration. Bar: 50 µm. **D** FISH. Liver section of seal PV20010801. No FISH signal within the necrotic areas, positive staining within individual adjacent hepatocytes (arrows). Bar: 20 µm.

Liver, spleen, lung, kidney, and brain samples of the ten RT-PCR hepatovirus-positive seals were investigated by histology and FISH. A positive RNA signal by FISH was observed in the liver tissues of seven of these seals. Histological analyses demonstrated morphologically normal hepatocytes in six seals (Fig. 3A) and areas of multifocal liver necrosis in four seals (Fig. 3C). Individual animals displayed either a mild, lymphohistiocytic and lymphoplasmacellular portal or a multifocal granulomatous and eosinophilic inflammation. By FISH it was observed that abundant seal hepatovirus RNA was present in the cytoplasm of morphologically normal hepatocytes (Fig. 3B). No seal hepatovirus RNA could be detected within the necrotic areas, but rather in healthy hepatocytes surrounding the lesion (Fig. 3D). Co-infection with PhHV-1 was detected by PCR screening of liver tissues from all animals displaying hepatic necrosis. No RNA signal was detected in tissue sections of liver from a PCR-negative seal. Detailed histological results for all tissues of seal hepatovirus-positive animals are summarized in Table S2.

### Serology

**Fig. 4:**
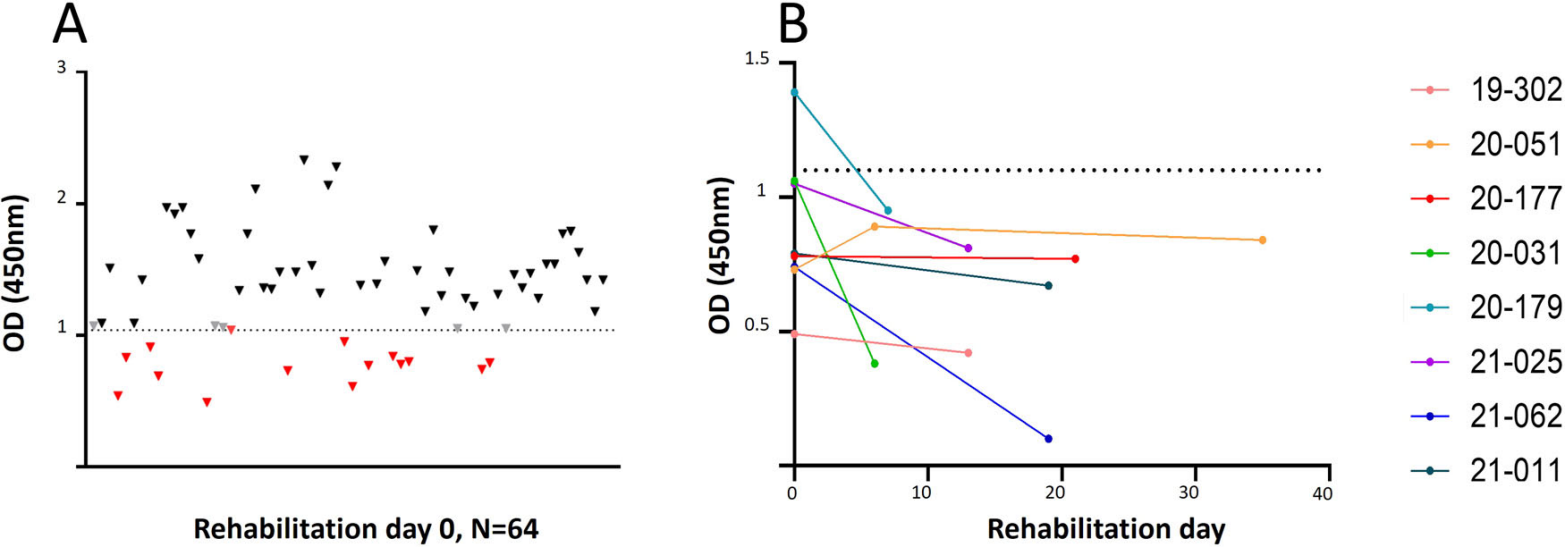
Serological screening of seal serum samples using a HAV-based ELISA. **A** OD from individual serum samples collected at rehabilitation day 0 (*n* = 64). Red: positive sera; black: negative sera; grey: indeterminate results. **B** OD from serum samples collected from animals at multiple days during the rehabilitation period. Dashed lines indicate the cutoff between positive and negative serum samples (OD = 1.04).

An HAV-based ELISA has been previously used to test serum samples from alpacas for the presence of antibodies against alpaca-HAV^8^, since antigenic determinants associated with blocking of hepatovirus-host cell attachment appear to be conserved between animal and human hepatoviruses^1^. We used the same serological assay for testing the seal sera, after confirming that 44 out of the 51 amino acids present in the previously identified HAV neutralization epitopes were also present in structural proteins of SealHAV_NL/PV/21 (Table S5). Seal serum samples were defined as positive when the OD value was lower or equal to 1.04, and as indeterminate when OD ranged between values 1.05 and 1.09. The mean OD of negative serum samples (1.514, SD = 0.3102) was significantly higher than that of positive serum samples (0.7165, SD = 0.2085) according to Mann–Whitney test (*P* < 0.0001) (Fig. S2). Hepatovirus-specific antibodies were detected in sera obtained from 14 juvenile harbor seals and one juvenile grey seal from a total of 64 animals (23%) at the day of admission to the Sealcentre Pieterburen (Fig. 4A). Serum samples from six out of ten animals that were positive for seal hepatovirus infection by RT-PCR also tested positive by ELISA. Thirteen serum samples from 8 harbor seals tested positive by HAV-ELISA at different time points during the rehabilitation period. A drop in OD, probably indicating an active or recent infection, was observed in five animals, and for one serum sample on day 7 following a negative OD reading on the day of admission (Fig. 4B; see Table S6). No clear macroscopic abnormalities related to liver disease could be observed in seropositive seals.

## Discussion

Several viruses that have caused major disease outbreaks in marine mammals have been identified in recent decades, including avian influenza-, herpes- and morbilli viruses^22–24^. With the advent of metagenomic sequencing methods, previously unknown viruses have also been identified in marine mammals, that did not cause overt clinical signs associated with the infection at the time of their detection. These included a parvo- and picornaviruses in seals as well as a pestivirus in porpoises^25–27^. The discovery of a novel hepatovirus in harbor and harp seals along the New England coast (USA) extended the known host range of hepatoviruses beyond humans and non-human primates^9^. These animals had died with an avian influenza A virus H3N8 infection. Thus far, little is known about geographical distribution, strain variation, pathogenesis, and prevalence of seal hepatovirus. Here, we describe a seal hepatovirus strain (SealHAV_NL/PV/21) infecting seals in coastal waters of The Netherlands. The virus was first detected by HTS in the liver tissue of a young harbor seal that died in a seal rehabilitation centre with PhHV-1 infection and hepatic necrosis. FISH results confirmed the presence of abundant seal hepatovirus RNA in the cytoplasm of histologically normal hepatocytes, while no hepatovirus RNA was detected in the necrotic lesion. Further analyses showed that the 4 SealHAV_NL/PV/21 infected seals with histopathological evidence of necrotic hepatitis were also co-infected with PhHV-1, which is an etiologic agent of necrotic hepatitis in seals^15^. In other animals, hepatitis characterized by the presence of eosinophils and granulomas suggested a bacterial or parasitic liver infection. These results indicate that, although abundantly present, the seal hepatovirus alone may not cause acute pathological changes in the liver of RT-PCR-positive juvenile animals, as has been also previously observed for seal hepatovirus-infected seals on the New England coast^9^ and for other small mammal and alpaca hepatoviruses^6,7^. Further molecular and serological screening to determine the prevalence of seal hepatovirus revealed endemic infections among the seals in this region. It remains to be determined to what extent seal hepatoviruses may be more pathogenic for adult seals.

The complete SealHAV_NL/PV/21 genome has 95.6% genome nucleotide identity with the seal hepatovirus strain previously identified in New England^9^. Full-length CDS-based phylogeny showed that seal hepatoviruses form a monophyletic group clustering with bat hepatoviruses (GenBank accession no. OQ818337 and no. NC038316). It is possible that harp seals could have acted as carrier species responsible for seal hepatovirus transmission between harbor seals in North America and Europe, as has also been speculated for initiation of the phocine distemper virus outbreak in 1988^26^. Comparison between SealHAV_NL/PV/2021 and New England seal hepatovirus 5′ UTR nucleotide sequences showed identical type III IRES sequences. This seal hepatovirus untranslated region retained a similar sequence and secondary structure as that of HAV type III IRES, involved in ribosome recruiting and translation initiation^9,28^. This suggests conservation of the same functions between human and seal hepatoviruses. Of note, in the 5′ UTR of SealHAV_NL/PV/2021, an additional 51 contiguous base pairs were observed, as compared to the one of New England virus. These repetitive sequences could have a role in influencing the rate of translation of the SealHAV_NL/PV/2021 proteins as compared to the New England virus forming additional RNA secondary structure.

Sequence analysis of the VP1 capsid protein-encoding gene sequences (831 bp) revealed minor genetic variation among seal hepatoviruses present infecting the ten positive harbor seals. This contrasts with the findings by Anthony et al., who reported 100% indentity in smaller VP1 amplicons (157 bp)^9^. Previous studies on HAV have also reported co-circulation of multiple genetic variants within a restricted geographic area^29,30^.

Retrospective serological analysis by a HAV-based competition ELISA revealed that 9.9% of animals (14 juvenile harbor seals, one juvenile grey seal) had seal hepatovirus antibodies on the day of admission to the Sealcentre Pieterburen. The positive samples also included six serum samples from RT-qPCR positive seals. Interestingly, we observed seroconversion (reduction in OD) of serum samples collected from five animals at different rehabilitation days at the Sealcentre. Among these, the serum sample obtained from PV21010601 (corresponding to rehab ID 20-179) tested positive at rehabilitation day 7, after having tested negative at the day of entry. These five cases indicate seal hepatovirus infections in animals shortly before or after admission to the Sealcentre.

It is interesting to note that the hepatovirus infected animals were all juvenile. The mild clinical picture and absence of lesions upon seal hepatovirus infection in the young seals is reminiscent of subclinical HAV infections in children^31^. Co-infections with PhHV-1 and seal hepatovirus in the young seals could have increased the likelihood of secondary bacterial infections, lungworm proliferation, and unresponsiveness to medications, necessitating their euthanasia. Consequently, serological testing during quarantine in the seal centre, and subsequent isolation would be an option to prevent spreading of the virus during their rehabilitation period. To what extent seal hepatoviruses may have long-term effects on the health status of the infected juvenile animals remains to be studied.

In summary, a new strain of seal hepatovirus has been identified in harbor seals in the North Sea and has 95.6% nucleotide identity to the seal hepatovirus strain previously discovered in seals in New England (USA) coastal waters. Retrospective molecular and serological data indicate that seal hepatovirus is enzootic in the seal population of the North Sea area, causing quiescent infections in young animals. Serological evidence indicates that the host range most likely extends to grey seals.

## Supplementary information

ARRIVE Compliance Questionnaire

Supplementary material

## Data Availability

The data that support the findings of this study are available on request from the corresponding author (A.O.).
