# Supplementary material for: Hepatovirus infections in juvenile seals from the North Sea
Source: Npj Viruses. 2025 Jan 8;3:1. doi: 10.1038/s44298-024-00084-8 (PMC11721073; doi:10.1038/s44298-024-00084-8)
Supplement: Supplementary file 1 — ARRIVE Compliance Questionnaire [file 44298_2024_84_MOESM1_ESM.pdf]

The ARRIVE Essential 10: Compliance Questionnaire

Use this questionnaire to evaluate how well a manuscript complies with the ARRIVE Essential 10. It can be applied to any manuscript describing comparative experiments in living animals, by assessors such as journal staff, editors, or peer reviewers.

Item	Question(s)	Answers
1 Study Design	Are all experimental and control groups clearly identified? This was not an intervention study with an experimental and a control animal group, but a clinical virus discovery study, within a well-defined animal clinical setting.	☐ Yes, for at least one experiment<br>☒ No
	Is the experimental unit (e.g. an animal, litter or cage of animals) clearly identified? Although this is not an experimental unit, all relevant environmental and clinical parameters are well defined or monitored	☒ Yes, for at least one experiment<br>☐ No
2 Sample Size	Is the exact number of experimental units in each group at the start of the study provided (e.g. in the format '*n*=')?	☒ Yes, for at least one experiment<br>☐ No In this study clinical and post-mortem data were collected within an animal clinical setting
	Is the method by which the sample size was chosen explained?	☐ Yes, for at least one experiment<br>☒ No Sample size of infected and non-infected animals was determined by the clinical and pathology findings
3 Inclusion & Exclusion Criteria	Are the criteria used for including and excluding animals, experimental units, or data points provided?	☒ Yes, for at least one experiment<br>☐ No Again, no experimental groups, but a clinical virus discovery study collecting clinical and post-mortem data within an animal clinical setting
	Are any exclusions of animals, experimental units, or data points reported, or is there a statement indicating that there were no exclusions?	☐ Yes, for at least one analysis<br>☒ No<br>The whole group in rehabilitation was evaluated
4 Randomisation	Is the method by which experimental units were allocated to control and treatment groups described?	☐ Yes, for at least one experiment<br>☒ No Clinical virus discovery study, within a well-defined animal clinical setting: observational taking all clinical, pathological and virological data into account
5 Blinding	Is it clear whether researchers were aware of, or blinded to, the group allocation at any stage of the experiment or data analysis? Initial clinical, pathology and viral investigations were carried out independently by the respective expertise centra	☒ Yes, for at least one experiment<br>☐ No
6 Outcome Measures	For all experimental outcomes presented, are details provided of exactly what parameter was measured?	☒ Yes, for at least one experiment<br>☐ No All parameters measured are presented
7 Statistical Methods	Is the statistical approach used to analyse each outcome detailed? Statistics were used in the context of the serological test to evaluate the sensibility of the assay used to test all serum samples	☒ Yes, for at least one analysis<br>☐ No
	Is there a description of any methods used to assess whether data met statistical assumptions? A normality test was done before applying the statistical test	☒ Yes, for at least one analysis<br>☐ No<br>☐ Not applicable
8 Experimental Animals	Are all species of animal used specified?	☒ Yes, for at least one experiment<br>☐ No We studied harbor seals and grey seals (Table S1)
	Is the sex of the animals specified?	☒ Yes, for at least one experiment<br>☐ No In Table S1, the sex is specified for each animal<br>☐ Not applicable to species
	Is at least one of age, weight or developmental stage of the animals specified?	☒ Yes, for at least one experiment<br>☐ No In table S1, it is specified is animals are pups, juveniles, or adults
9 Experimental Procedures	Are both the timing and frequency with which procedures took place specified?	☒ Yes, for at least one experiment<br>☐ No Clinical, pathology and virological data are all related to time(s) of sampling
	Are details of acclimatisation periods to experimental locations provided?	☐ Yes, for at least one experiment<br>☒ No Standardized quarantining is practiced for incoming animals
10 Results	Are descriptive statistics for each experimental group provided, with a measure of variability (e.g. mean and SD, or median and range)?	☒ Yes, for at least one experiment<br>☐ No<br>☐ Not applicable to the type of data collected
	Is the effect size and confidence interval provided?	☐ Yes, for at least one experiment<br>☒ No<br>☐ Not applicable to the type of analysis used

Notes on questionnaire design

The ARRIVE guidelines are a useful resource for authors preparing manuscripts describing animal research, and also provide a framework to evaluate the transparency of those manuscripts. To assess reporting quality, numerous studies have in the past sought to operationalise reporting guidelines (including ARRIVE). Typically, this involves scoring a manuscript's degree of compliance with guideline items in a binary fashion (e.g. an item is either not reported or reported) [1-3], a graded fashion (e.g. not, partially, or completely reported) [4,5], or a combination of the two [6].

This questionnaire has been designed to be as concise and user-friendly as possible. The number of questions used to assess a manuscript's compliance has been kept to a minimum, and in most cases each question is designed to be answered in a binary fashion. Compliance with some Essential 10 sub-items is inherently impossible to judge in this way, instead requiring a subjective judgement on the level of detail provided. For this reason, not all sub-items are represented by a question in this questionnaire.

To facilitate binary answers, it has been necessary to identify the minimum information in a manuscript sufficient to comply with each question. The strengths of this approach include the relatively short length of the questionnaire (and the correspondingly low time burden of using it), and the avoidance of ambiguity that would arise from a graded answering system, in which an intermediate score (e.g. 'partially/insufficiently reported') could denote a number of distinct deficiencies in compliance with an item (e.g. either only part of the item was complied with, or only the reporting of some experiments in the manuscript complied with the item.)

Limitations of this approach centre on the necessity to identify the minimum information sufficient to comply with each question. In some cases, this has resulted in questions that require a guideline sub-item's criteria to have been fulfilled in the reporting of only one experiment in a manuscript. As a result, not all experiments in a manuscript may be described in a way that fulfils that criterion, despite the manuscript being considered to comply with the guidelines overall.

References

1. Hair *et al* (2020). *Res Integ Peer Rev*. doi: 10.1186/s41073-019-0069-3
2. Tihanyi *et al* (2019). *J Surg Res*. doi: 10.1016/j.jss.2018.10.038
3. Zhao *et al* (2020). *BMC Vet Res*. doi: 10.1186/s12917-020-02664-1
4. Han *et al* (2017). *Plos One*. doi: 10.1371/journal.pone.0183591
5. Chatzimanouil *et al* (2019). *J Am Soc Nephrol*. doi: 10.1681/ASN.2018050515
6. Leung *et al* (2018). *Plos One*. doi: 10.1371/journal.pone.0197882
