# Supplementary material for: Hepatovirus infections in juvenile seals from the North Sea
Source: Npj Viruses. 2025 Jan 8;3:1. doi: 10.1038/s44298-024-00084-8 (PMC11721073; doi:10.1038/s44298-024-00084-8)
Supplement: Supplementary file 2 — Supplementary material [file 44298_2024_84_MOESM2_ESM.pdf]

Table S1: Details on screened seals and carcass preservation status at the necropsy

External_ID (Ursprung)	Rehab ID	Carcass at necropsy	State of autolysis	Specie (PV /HG)	Sex (M/F)	Estimated age (pup, juvanile, adult)	Age estimated
HG20022301		cooled	1	Grey seal	Male	Juvenile	2 or 3 months
HG20022601	20-039	cooled	1	Grey seal	Male	Juvenile	3 months
HG20031301	20-057	defrost	1	Grey seal	Male	Juvenile	4 months
HG20031601	20-058	cooled	1	Grey seal	Female	Juvenile	4 months
HG20090501	20-063	cooled	1	Grey seal	Male	Juvenile	4 months
PV20010401		cooled	1	Harbor seal	Male	Juvenile	7 months
PV20010801	19-302	cooled	1	Harbor seal	Female	Juvenile	7 months
PV20020301	20-019	cooled	1	Harbor seal	Female	Juvenile	8 months
PV20021501	20-031	cooled	1	Harbor seal	Female	Juvenile	8 months
PV20021701		fresh	1	Harbor seal	Female	Adult	3 years
PV20031101	20-055	fresh	1	Harbor seal	Female	Juvenile	9 months
PV20070201	20-099	defrost	1	Harbor seal	Female	Pup	2 weeks
PV20070301	20-109	defrost	1	Harbor seal	Male	Pup	3- 4 days
PV20070601	20-091	defrost	2	Harbor seal	Female	Pup	3- 4 days
PV20070801		defrost	3	Harbor seal	Male	Pup	2- 4 days
PV20070802	20-125	cooled	1	Harbor seal	Male	Pup	12-15 days
PV20070901		defrost	1	Harbor seal	Male	Pup	7-10 days
HG20071201	20-078	defrost	1	Grey seal	Male	Juvenile	4 months
PV20071301	20-136	cooled	1	Harbor seal	Female	Pup	7-10 days
PV20071401		cooled	1	Harbor seal	Male	Pup	7-10 days
PV20071501		defrost	1	Harbor seal	Female	Juvenile	9 months
PV20071901		not given	1	Harbor seal	Male	Unreported	Unreported
PV20071902		defrost	2	Harbor seal	Male	Pup	10 days
PV20072001	20-051	defrost	2	Harbor seal	Male	Juvenile	9 months
PV20072101	19-279	defrost	2	Harbor seal	Male	Juvenile	5 months
PV20072301	20-145	cooled	1	Harbor seal	Male	Pup	1 month
PV20080201		cooled	1	Unreported	Unreported	Unreported	Unreported
PV20081601	20-133	cooled	1	Harbor seal	Male	Pup	1 month
PV20081701		Cooled	2	Harbor seal	Male	Adult	1 year
PV20082001	20-062	defrost	1	Harbor seal	Male	Juvenile	10 months

HG20082101		defrost	1	Grey seal	Male	Pup	1,5 months
HG20082201		defrost	2	Grey seal	Female	Juvenile	HG moulted weaner (~3 months)
PV20082701	20-148	fresh	1	Harbor seal	Male	Juvenile	2 months
PV20090401		Defrost	not given	Harbor seal	Male	Unreported	Unreported
PV20090502		cooled	1	Harbor seal	Male	Juvenile	3 months
PV20091601	20-153	cooled	1	Harbor seal	Female	Juvenile	3 months
PV20092401		cooled	1	Harbor seal	Male	Juvenile	3 months
PV20092501		cooled	1	Harbor seal	Female	Juvenile	3 months
PV20092901	20-152	cooled	1	Harbor seal	Male	Juvenile	3 months
PV20100301		cooled	1	Harbor seal	Male	Juvenile	3 months
PV20110701		cooled	1	Harbor seal	Male	Juvenile	5 months
PV20112201		cooled	1	Harbor seal	Female	Juvenile	5 months
PV20120801	20-171	cooled	1	Harbor seal	Male	Juvenile	6 months
PV20121401	20-172	cooled	1	Harbor seal	Female	Juvenile	6 months
PV20122901	20-178	cooled	1	Harbor seal	Female	Juvenile	6 months
HG21010701		fresh	1	Grey seal	Male	Pup	1 month
HG21021701	20-030	cooled	1	Grey seal	Male	Adult	1 year old
HG21052201		cooled	1	Grey seal	Female	Juvenile	6 months
HG21120601		cooled	1	Grey seal	Male	Pup	7-10 days
PV21010601	20-179	cooled	1	Harbor seal	Male	Juvenile	6 months
PV21011201	20-151	cooled	1	Harbor seal	Male	Juvenile	7 months
PV21011301	21-009	cooled	1	Harbor seal	Male	Juvenile	7 months
PV21011501	20-177	cooled	1	Harbor seal	Male	Juvenile	7 months
PV21012401	20-003	fresh	1	Harbor seal	Male	Juvenile	7 months
PV21013001		cooled	1	Harbor seal	Female	Juvenile	7 months
PV21020201	21-011	not given	1	Harbor seal	Female	Juvenile	8 months
PV21021001	21-026	cooled	1	Harbor seal	Male	Juvenile	8 months
PV21021301	21-027	cooled	1	Harbor seal	Female	Juvenile	8 months
PV21022101	21-025	cooled	1	Harbor seal	Female	Juvenile	8 months
PV21022102	21-036	cooled	1	Harbor seal	Female	Juvenile	8 months
PV21022801	21-037	fresh	1	Harbor seal	Male	Juvenile	8 months
PV21041401	21-056	cooled	1	Harbor seal	Male	Juvenile	10 months

PV21042101	21-043	cooled	1	Harbor seal	Male	Juvenile	10 months
PV21042801	21-050	cooled	1	Harbor seal	Female	Juvenile	10 months
PV21050201		cooled	1	Harbor seal	Female	Juvenile	10 months
PV21050701		cooled	1	Harbor seal	Male	Adult	2-4 years
PV21051101	21-062	fresh	1	Harbor seal	Female	Juvenile	11 months
PV21051301		cooled	1	Harbor seal	Male	Pup	Premature pup
PV21053001		cooled	1	Harbor seal	Female	Pup	7-10 days (premature)
PV21060501		cooled	1	Harbor seal	Female	Unreported	Unreported
PV21060901		not given	3	Harbor seal	Female	PupuPup	0-1 days
PV21061801	21-078	cooled	1	Harbor seal	Male	Pup	5-6 days
PV21070201		cooled	2	Harbor seal	male	PupuPup	7-10 days
PV21070501	21-094	cooled	1	Harbor seal	Male	Pup	20 days
PV21070502	21-110	cooled	1	Harbor seal	Female	Pup	15 days
PV21070701	21-098	cooled	1	Harbor seal	Female	Pup	Not given
PV21070702	21-105	cooled	1	Harbor seal	Male	Pup	15-20 days
PV21072501	21-120	cooled	1	Harbor seal	Male	Pup	1 month
PV21072801		fresh	1	Harbor seal	Male	Pup	1 month
PV21080201		fresh	1	Harbor seal	Female	Pup	1 month
PV21082801		fresh	1	Harbor seal	Unreported	Pup	2 month
PV21090101	21-126	fresh	1	Harbor seal	Female	Juvenile	2 months
PV21090901	21-128	fresh	1	Harbor seal	Male	Juvenile	3 months
PV21091701		cooled	1	Harbor seal	Male	Juvenile	3-4 months
PV21092601	21-123	fresh	1	Harbor seal	Female	Juvenile	3 months
PV21092901	21-134	cooled	1	Harbor seal	Male	Juvenile	3 months
PV21101501	21-136	cooled	1	Harbor seal	Female	Juvenile	4 months
PV21101801		cooled	1	Harbor seal	Male	Juvenile	4 months
PV21102001		cooled	1	Harbor seal	Female	Juvenile	4 months
PV21102401	21-146	cooled	1	Harbor seal	Female	Juvenile	4 months
PV21102501	21-138	cooled	1	Harbor seal	Female	Juvenile	4 months
PV21102601	21-148	cooled	1	Harbor seal	Female	Juvenile	4 months
PV21110101		cooled	1	Harbor seal	Male	Juvenile	4 months
PV21111001		cooled	1	Harbor seal	Female	Juvenile	5 months

PV21111301		cooled	1	Harbor seal	Male	Adult	3 years
PV21112001	21-153	cooled	1	Harbor seal	Female	Juvenile	5 months
PV21112101	21-161	cooled	1	Harbor seal	Female	Juvenile	5 months
PV21112201		fresh	1	Harbor seal	Male	Adult	not specified
PV21120401	21-143	defrost	1	Harbor seal	Female	Juvenile	4 months
PV21120801		defrost	3	Harbor seal	Male	Pup	10 days
PV21120901	21-158	cooled	1	Harbor seal	Male	Pup	10 days

Table S2: Cause of death and histological lesions present in tissues of seal hepatovirus RT-PCR-positive harbor seals.

Animal ID	Rehab ID	Species	Gender	Life stage	COD and main gross-pathological findings	Histopathological findings
PV20010401	No rehab ID available°	harbor seal	male	7 months	COD: enthanasia, massive lungworm infestation and emphisema. Lung: marble pattern; pus in bronchioli and parenchyma; llw; slw; liver: punctuate white spots; pale; spleen: pale; kidney: congested; brain: congested;	Lung: moderate, chronic-active, suppurative bronchopneumonia with atelectasis; moderate, pyo-granulomatous Pnuemonia and intralesional nematodes; liver: moderate, multifocal pyo-granulomatous and eosinophilic hepatitis with moderate capsular fibrosis; spleen: moderate plasmocytosis and pigment storage in macrophages; mild extramedullary hematopoiesis; kidney: NAD; brain: mild, multifocal granulohistiocytic and eosinophilic vasculitis and perivascular encephalitis with mild hemorrhage;
PV20010801*	19-302	harbor seal	female	7 months	COD: Septicemia. Lung: marble pattern; interstitial emphysema; lungworm; liver: pale color; mf, spot-like, white lesions on capsule; spleen: enlarged; granulated texture; kidneys: prominent whitish structure on capsule; cerebrum: susp. of edema	Lung: individual bronchial epithelial cells with eosinophilic inclusion bodies; multifocal atelektasis; liver: moderate to severe, disseminated, acute, necrotizing hepatitis with eosinophilic to amphophilic intranuclear inclusion bodies; spleen: mild to moderate hemosiderosis and mild extramedullary hematopoiesis; mild, disseminated single-cells necrosis; kidney: NAD; brain: NAD
PV20072101	19-279	harbor seal	male	5 months	COD: enthanasia. Lung: both sides enlarged, compact with mf, pale, hard nodules; hemorrhagic pneumonia with lLW and sLW; emphysema; liver: mf pale areas; spleen: NAD; kidneys: NAD; brain: NAD	Lung: mild, multifocal, interstitial, lymphoplasmacellular pneumonia; multifocal, moderate granulohistiocytic and eosinophilic and partially necrotizing pneumonia with intralesional nematodes; mild alveolar and interstitial edema; liver: NAD; spleen: NAD kidney: NAD; brain: not available for histolopathological investiagtion;
PV20120801	20-171	harbor seal	male	~ 6 months	COD: enthanasia. Lung: compact parenchyma, heterogenous color; hemorrhages; interstitial emphysema; lLW; sLW; atelectasis; edema; liver: compact parenchyma; mf fibrotic nodules; spleen: NAD; kidneys: NAD; brain: NAD	Lung: mild, multifocal, lymphohistiocytic bronchitis with mild proliferation of vascular intima of individual arteries; moderate endoparasitosis; liver: mild, multifocal, lymphohistiocytic, partially granulomatous and eosinophilic hepatitis; spleen: mild to moderate depletion of white pulp; kidney: NAD; brain: NAD
PV20121401	20-172	harbor seal	female	~ 6 months	COD: enthanasia. Lung: redding and compact parenchyma; interstitial emphysema; haemorrhagic bronchopneumonia; lLW; edema; liver: multiple capsular fibrotic nodules; spleen: NAD; kidneys: NAD; brain: NAD	Lung: mild, multifocal, lymphohistiocytic , interstitial pneumonia with mild interstitial fibrosis; mild, suppurative bronchiolitis; mild, multifocal endoparasitosis with associated atelektasis; local thrombus in a single artery; liver: mild, multifocal, portal, lymphoplasmacellular hepatitis and mild to moderate lymphohistiocytic hepatitis with individual hepatocyte loss; moderate, focal, granulomatous hepatitis; spleen: mild plasmocytosis and mild, follicular hyperplasia; mild extramedullary hematopoiesis; kidney: mild, focal, lymphoplasmacellular, interstitial nephritis with mild, interstitial fibrosis; brain: NAD;
PV21010601*	20-179	harbor seal	male	~ 6 months	COD: severe, generalized, bilateral interstitial pneumonia. Lung: enlarged, no collapse; patchy redding; focal consolidation; interstitial edema; mf hemorrhage and necrosis; liver: pale; lobular pattern; oligofocal white, hard lesions with hemorrhagic cavities; spleen: NAD; kidneys: NAD; brain: NAD Liver: multifocally necrotizing and granulomatous hepatitis. Moderate numbers of lymphocytes, plasma cells and macrophages. Presence of mild margination of the nucleus with indications for intranuclear viral inclusions.	Lung: chronic-active, lymphohistiocytic and granulocytic bronchopneumonia and necrotizing bronchitis with epithelial hyperplasia; eosinophilic intranuclear inclusion bodies; multifocal, granulomatous to lymphoplasmacellular pneumonia with intralesional nematodes; liver: multifocal to coalescing, acute to subacute, necrotizing hepatitis with eosinophilic intranuclear inclusion bodies; spleen: NAD; kidney: NAD; brain: NAD
PV21011301	21-009	harbor seal	male	~ 7 months	COD: hypoxia caused by a severe interstitial pneumonia. Lung: enlarged; patchy redding; consolidated areas; interstitial emphysema; lLW; Pus in bronchi; liver: miliary white spotson capsule and within the parenchyma; mf pale areas; lobular pattern; spleen: NAD; kidneys: NAD; brain: NAD	Lung: mild to moderate, multifocal, lymphoplasmacellular, interstitial pneumonia and mild, suppurative bronchopneumonia, associated with lobular atelektasis; mild, mild, focal, pyo-granulomatous pneumonia with intralesional nematodes; liver: mild, multifocal, lymphohistiocytic to pyo-granulomatous and eosinophilic hepatitis; spleen: mild plasmocytsosi and extramedullary hematopoiesis; kidney: NAD; brain: NAD
PV21011501*	20-177	harbor seal	male	~ 7 months	COD: Hepatic failure Lung: enlarged; not collapsed; patchy redding; areas of haemorrhage and necrosis; liver: nutmeg pattern; spleen: NAD; kidneys: NAD; brain: NAD	Lung: mild, multifocal, lymphoplasmacellular peribronchitis and mild endoparasitosis; liver: severe, multifocal to coalescing, granulohistiocytic hepatitis with necrosis and hemorrhage; eosinophilic to amphophilic, intranuclear inclusion bodies; focal, portal fibrosis and cholestasis; spleen: NAD; kidney: NAD; brain: NAD
PV21020201*	21-011	harbor seal	female	~ 8 months	COD: euthanasia. Lung: enlarged; mild marble pattern; interstitial pneumonia; edema; interstitial emphysema; liver: pale; friable; nutmeg pattern; mf fibrotic nodules; spleen: pale; white pulp hyperplasia; kidneys: congested; brain: congested	Lung: multifocal, granulomatous pneumonia with necrosis and intralesional nematodes; severe interstitial emphysema; eosinophilic intranuclear inclusion bodies; liver: disseminated, acute, necrotizing hepatitis with eosinophilic intranuclear inclusion bodies; spleen: NAD; Kidney: mild, multifocal, lymphoplasmacellular, interstitial nephritis; brain: NAD
PV21120901	21-158°	Harbor seal	Male	10 days	COD: euthanasia. (Possible sepsis / No significant liver lesions)	Lung: mild, multifocal, lymphoplasmacellular, interstitial pneumonia; moderate endoparasitosis with associated atelektasis and focal granulomatous and eosinophilis pneumonia; liver: moderate, focal, granulomatous and eosinophilic hepatitis with intralesional nematodes; spleen: mild, follicular hyperplasia with moderate plasmocytosis and mild hemosiderosis; kidney: NAD; brain: NAD

*phocid herpesvirus 1 co-infection
Notes: COD (Cause Of Death); NAD (No Abnormality Detected)
°serum not available for this animal

Table S3: Synonimus mutations observed in the VP1 gene of the phopivirus present in infected animals

	VP1 gene position							
**Sample external ID**	57	84	147	192	240	408	483	822
PV20010401*								
PV20010801						U/A		
PV20072101								
PV20120801			A/G	A/G	U/C			A/G
PV20121401	U/C							
PV21010601			A/G	A/G	U/C			A/G
PV21011301			A/G	A/G	U/C			A/G
PV21011501			A/G	A/G	U/C			A/G
PV21020201			A/G	A/G	U/C			A/G
PV21120901	U/C	U/C					U/C	

*Mutations are reported as compared to PV20010401 seal hepatovirus VP1 gene upon nucleotide pairwise alignment

Table S4: Virus sequences used for phylogenetic tree construction.

Genbank accession number	Hepatovirus strain name	
AB258387	Hepatitis A virus genomic RNA subgenotype IIIB	
AB279732	Hepatitis A virus	
AB279733	Hepatitis A virus isolate: HA-JNG08-92	
AB279734	Hepatitis A virus isolate HAJ95-8	
AB279735	Hepatitis A virus genomic RNA isolate: HAJ85-1	
AB300205	Hepatitis A virus sub strain: KRM238G59	
AB425339	Hepatitis A virus sub strain: KRM003G72	
AB618529	Hepatitis A virus isolate: HAJIH-Fukuo10	
AB618531	Hepatitis A virus isolate: HAJNS-BorSap10	
AB623053	Hepatitis A virus isolate: HA286-Aki1957	
AB839692	Hepatitis A virus isolate: BaliA03-H29	
AB839693	Hepatitis A virus isolate: JemberA07-SBY07	
AB839694	Hepatitis A virus: MakassarA07-R18	
AB839695	Hepatitis A virus isolate: MataramA07-RS03	
AB839696	Hepatitis A virus isolate: SoloA07-P15	
AB839697	Hepatitis A virus isolate: TangerangA07-55	
**AJ225173.1**	**Avian encephalomyelitis virus mRNA**	**Outgroup**
BR001716.1	Goat hepatovirus goat/2019/1	
EU011791	Hepatitis A virus isolate PN-IND	
FJ360735	Hepatitis A virus isolate IND-HAV-97F	
HM769724	Hepatitis A virus isolate HAV-Arg/06	
JQ655151	Hepatitis A virus isolate Kor-HAV-F	
KT452695	Hedgehog hepatovirus isolate Igel75Erieur2014	
KT452698.1	Hedgehog hepatovirus isolate Igel68Erieur2014	
KT877158	Tupaia hepatovirus A	
KY003229	Hepatovirus A	
LC435031	Human hepatitis A virus HA18-1053	
LC515201	Hepatovirus A SYMAV-D12/Gabon/2016	
MG049743	Hepatovirus A strain Sao Paulo	
MG181943.1	Didelphis aurita hepatitis A	
MH577311	Hepatovirus A strain USA/2018/V18S00294	
MH577312	Hepatovirus A strain USA/2017/V17S07512	
MN062166	Hepatovirus A strain USA/2018/V18S01071	
MN062167	Hepatovirus A strain USA/2018/V18S02170	
NC 028363.1	Rodent hepatovirus RMU101637Micarv2010	
NC 028365.1	Hedgehog hepatovirus Igel8Erieur2014	
NC 028981.1	Tupaia hepatovirus A	
NC 038313.1	Bat hepatovirus SMG18520Minmav2014	
NC 038314.1	Rodent hepatovirus CIV459Lopsik2004	
NC 038316.1	Bat hepatovirus BUO2BF86Colafr2010	
NC027818.1	seal hepatovirus NewEngland_USA/2011	
OK625565	Hepatovirus A isolate Hepatovirus A/0789/Haiti/2016	
OM302498.1	Eptesicus fuscus hepatovirus isolate 15893	
ON524514	Hepatovirus A strain HepA/USA NY/182789/2020	
ON524530	Hepatovirus A strain HepA/USA NY/42769/2021	
OR452343.1	Hepatovirus A isolate Chu-alp-20	
OR452344.1	Hepatovirus A isolate Chu-alp-21	

Table S5: Hepatovirus epitopes associated with neutralization in the structural proteins.

Virus name	Homology among domains associated with anti-HAV mAB binding							
	**VP2**		**VP3**				**VP1**	
	64-71	68-78	102-121	143-150	209	246*	102	171
**SealHAV_NL/PV/21**	HTAEWTTT	ASQTVGTQIKV	ASISQMFCFWRGDLVFDFQV	DVSKVTLK	R	Q	N	V
**HAV Gen bank acc.# M14707**	HSADWLTT	ASDSVGQQIKV	ASISQMFCFWRGDLVFDFQV	DVSGITLK	R	Q	N	V

In red: amino acids in HAV that differ from SealHAV_NL/PV/21 in the same genome position

Table S6: Individual serological test results.

Species	Animal ID number	Rehab ID	Rehab day	OD	Comments
Harbor seal	NA	21-165	0	1.07	Animal was released
Harbor seal	PV21122501	21-166	0	1.09	
Harbor seal	PV22011502	21-167	0	1.51	
Harbor seal	NA	21-168	0	0.54	Animal was released
Harbor seal	NA	21-169	0	0.83	Animal was released
Harbor seal	NA	21-170	0	1.09	Animal was released
Harbor seal	NA	21-171	0	1.42	Animal was released
Harbor seal	NA	21-172	0	0.91	Animal was released
Harbor seal	NA	21-173	0	0.69	Animal was released
Grey seal	NA	22-022	0	1.97	Animal was released
Grey seal	HG20022601	20-039	0	1.92	
Grey seal	HG20031301	20-057	0	1.97	
Grey seal	HG20031601	20-058	0	1.77	
Grey seal	HG20090501	20-063	0	1.58	
Harbor seal	PV20010801	19-302	0	0.49	
Harbor seal	PV20020301	20-019	0	1.07	
Harbor seal	PV20021501	20-031	0	1.06	
Harbor seal	PV20031101	20-055	0	1.04	
Harbor seal	PV20070201	20-099	0	1.34	
Harbor seal	PV20070301	20-109	0	1.77	
Harbor seal	PV20070601	20-091	0	2.11	
Harbor seal	PV20070802	20-125	0	1.36	
Harbor seal	PV20071201	20-078	0	1.35	
Harbor seal	PV20071301	20-136	0	1.48	
Harbor seal	PV20072001	20-051	0	0.73	
Harbor seal	PV20072101	19-279	0	1.48	
Harbor seal	PV20072301	20-145	0	2.33	
Harbor seal	PV20081601	20-133	0	1.53	
Harbor seal	PV20082001	20-062	0	1.32	
Harbor seal	PV20082701	20-148	0	2.14	
Harbor seal	PV20091601	20-153	0	2.28	
Harbor seal	PV20120801	20-171	0	0.95	
Harbor seal	PV20121401	20-172	0	0.61	
Harbor seal	PV20122901	20-178	0	1.38	
Harbor seal	NA	20-030	0	1.18	Animal was released
Harbor seal	PV21010601	20-179	0	1.39	
Harbor seal	PV21011201	20-151	0	1.56	
Harbor seal	PV21011301	21-009	0	0.84	
Harbor seal	PV21011501	20-177	0	0.78	
Harbor seal	PV21012401	20-003	0	0.80	
Harbor seal	PV20092901	20-152	0	1.49	
Harbor seal	PV21021301	21-027	0	1.18	
Harbor seal	PV21070701	21-098	0	1.80	
Harbor seal	PV21022102	21-036	0	1.30	
Harbor seal	PV21022801	21-037	0	1.48	
Harbor seal	PV21041401	21-056	0	1.05	
Harbor seal	PV21042101	21-043	0	1.28	
Harbor seal	PV21042801	21-050	0	1.22	
Harbor seal	PV21051101	21-062	0	0.74	
Harbor seal	PV21020201	21-011	0	0.79	
Harbor seal	PV21021001	21-026	0	1.31	
Harbor seal	PV21022101	21-025	0	1.05	
Harbor seal	PV21070702	21-105	0	1.46	
Harbor seal	PV21092901	21-134	0	1.36	
Harbor seal	PV21061801	21-078	0	1.47	
Harbor seal	PV21070501	21-094	0	1.28	
Harbor seal	PV21101501	21-136	0	1.54	
Harbor seal	PV21102401	21-146	0	1.54	
Harbor seal	PV21102501	21-138	0	1.77	
Harbor seal	PV21102601	21-148	0	1.79	
Harbor seal	PV21112001	21-153	0	1.63	
Harbor seal	PV21120401	21-143	0	1.42	
Harbor seal	PV21021701	21-030	0	0.77	
Harbor seal	PV21072501	21-120	0	1.42	
Harbor seal	PV20010801	19-302	13	0.42	
Harbor seal	PV20021501	20-031	16	0.38	
Grey seal	HG20022601	20-039	9	1.91	
Harbor seal	PV20072001	20-051	6	0.89	
Harbor seal	PV20072001	20-051	35	0.84	
Grey seal	HG20031601	20-058	5	1.55	
Harbor seal	PV20082001	20-062	18	1.67	
Grey seal	HG20090501	20-063	13	1.79	
Harbor seal	PV20070201	20-099	8	1.46	
Harbor seal	PV21011201	20-151	24	1.29	

Harbor seal	PV20092901	20-152	30	1.84	
Harbor seal	PV21011501	20-177	21	0.77	
Harbor seal	PV21010601	20-179	7	0.95	
Harbor seal	PV21020201	21-011	19	0.67	
Harbor seal	PV21022101	21-025	13	0.81	
Harbor seal	PV21022801	21-037	3	1.21	
Harbor seal	PV21042101	21-043	11	0.98	
Harbor seal	PV21051101	21-062	19	0.10	
Harbor seal	PV21061801	21-078	21	1.77	
Harbor seal	PV21070501	21-094	8	1.56	
Harbor seal	NA	21-095	9	1.79	Animal was released
Harbor seal	PV21072501	21-120	3	1.79	
Harbor seal	PV21101501	21-136	12	1.46	
Harbor seal	PV21102501	21-138	19	1.31	
Harbor seal	PV21112001	21-153	6	0.88	
Harbor seal	PV21120901	21-158	17	0.47	

NA= Not Available

NT= Not Tested

*Positive by RT-PCR

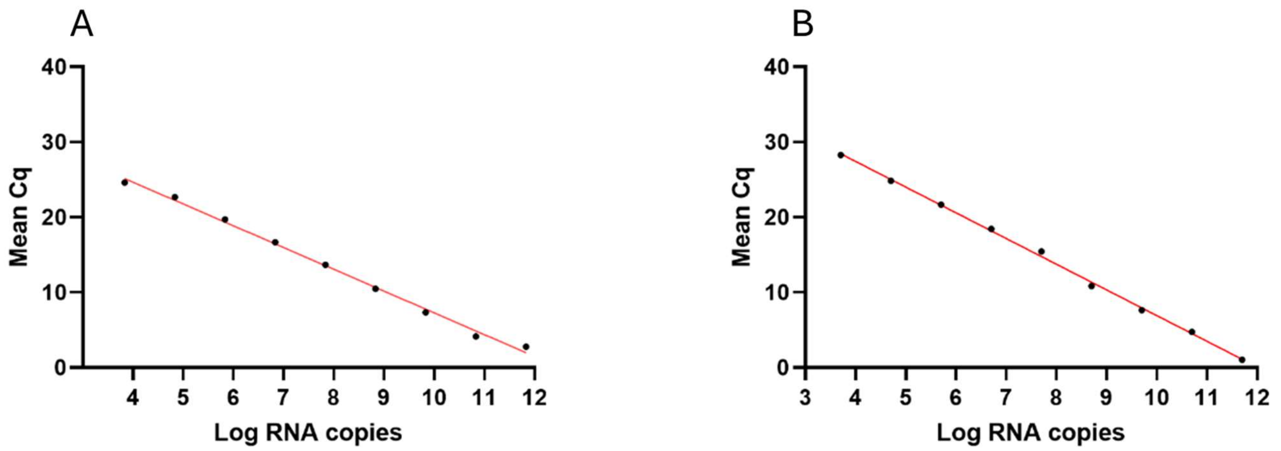

Figure S1: Amplification efficiency for a new Phopivirus specific RT-qPCR assay. Standard curves for positive Phopivirus control (A) and for heterologous internal EGFP control (B) Mean input RNA copy numbers from nine replicates of 10-fold dilutions are plotted against their mean Ct values. The linear regression area of the plot is shown. Linear regression analysis for panels A and B was performed using GraphPad Prism 8 (GraphPad Software Inc.).

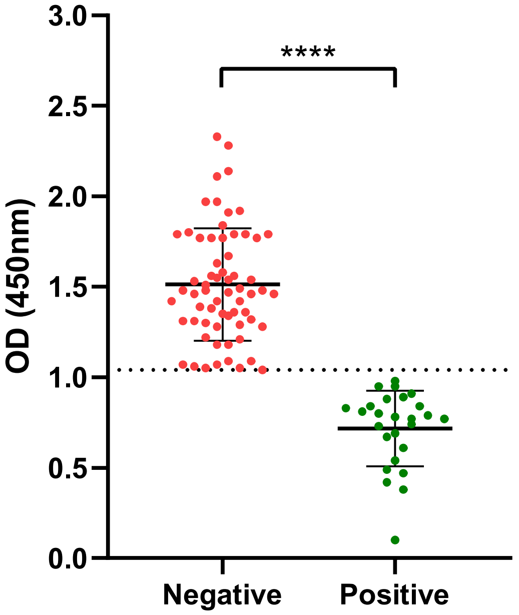

Figure S2: Optical density (OD) values of negative and positive seal serum samples tested by the HAV-ELISA. Individual OD values of negative (red dots) and positive (green dots) seal serum samples are reported together with group mean ± standard deviation. The dashed line represents the cut off used in the serological test to distinguish between positive and negative seal serum samples for HAV cross-reactive antibodies (OD=1,04). Results are reported as individual scores, n=90. Groups significantly differed ****p<0.0001.
